# Associations of Plasma Glutamatergic Metabolites with Alpha Desynchronization during Cognitive Interference and Working Memory Tasks in Asymptomatic Alzheimer’s Disease

**DOI:** 10.3390/cells13110970

**Published:** 2024-06-04

**Authors:** Vincent Sonny Leong, Jiaquan Yu, Katherine Castor, Abdulhakim Al-Ezzi, Xianghong Arakaki, Alfred Nji Fonteh

**Affiliations:** 1 Cognition and Brain Integration Laboratory, Neurosciences Department, Huntington Medical Research Institutes, Pasadena, CA 91105, USAxianghong.arakaki@hmri.org (X.A.); 2 Biomarker and Neuro-Disease Mechanism Laboratory, Neurosciences Department, Huntington Medical Research Institutes, Pasadena, CA 91105, USA

**Keywords:** pyroglutamate, glutamine, glutamate, electroencephalogram (EEG), Alzheimer’s disease (AD), event-related alpha desynchronization (ERD)

## Abstract

Electroencephalogram (EEG) studies have suggested compensatory brain overactivation in cognitively healthy (CH) older adults with pathological beta-amyloid(Aβ_42_)/tau ratios during working memory and interference processing. However, the association between glutamatergic metabolites and brain activation proxied by EEG signals has not been thoroughly investigated. We aim to determine the involvement of these metabolites in EEG signaling. We focused on CH older adults classified under (1) normal CSF Aβ_42_/tau ratios (CH-NATs) and (2) pathological Aβ_42_/tau ratios (CH-PATs). We measured plasma glutamine, glutamate, pyroglutamate, and γ-aminobutyric acid concentrations using tandem mass spectrometry and conducted a correlational analysis with alpha frequency event-related desynchronization (ERD). Under the N-back working memory paradigm, CH-NATs presented negative correlations (r = ~−0.74–−0.96, *p* = 0.0001–0.0414) between pyroglutamate and alpha ERD but positive correlations (r = ~0.82–0.95, *p* = 0.0003–0.0119) between glutamine and alpha ERD. Under Stroop interference testing, CH-NATs generated negative correlations between glutamine and left temporal alpha ERD (r = −0.96, *p* = 0.037 and r = −0.97, *p* = 0.027). Our study demonstrated that glutamine and pyroglutamate levels were associated with EEG activity only in CH-NATs. These results suggest cognitively healthy adults with amyloid/tau pathology experience subtle metabolic dysfunction that may influence EEG signaling during cognitive challenge. A longitudinal follow-up study with a larger sample size is needed to validate these pilot studies.

## 1. Introduction

Alzheimer’s Disease (AD) is a progressive brain disease characterized by significant memory loss and cognitive dysfunction. The main pathological features (beta-amyloid plaques, tau tangle accumulation, and synaptic dysfunction) occur before mild cognitive impairment (MCI) can be clinically diagnosed [1,2,3]. A neuropsychological evaluation of cognitively healthy older adults revealed color-word Stroop interference testing and the deterioration of core executive function performance as significant predictors of pathological status [2]. Of the core executive functions, working memory (WM) deficits are one of the first reported symptoms in early disease stages [4]. Beta-amyloid (Aβ_42_) and tau biomarkers can be detected in cerebrospinal fluid (CSF) to analyze early AD pathogenesis but are limited due to the invasiveness of lumbar punctures and its large margin of error for a definitive diagnosis [3,5,6,7]. Both symptomatic and presymptomatic AD patients display a poor correlation between the amount of Aβ_42_ plaques and the severity of cognitive impairment. However, pathological Aβ_42_/tau ratios remain commonly used to differentiate cognitively healthy (CH) patient cohorts due to its sensitivity in determining symptomatic AD [8,9,10,11]. Compared with CH individuals with normal CSF Aβ_42_/tau ratios (CH-NATs), CH individuals with pathological Aβ_42_/tau ratios (CH-PATs) are at a greater risk of cognitive deterioration to MCI or AD.

**Figure 1:**
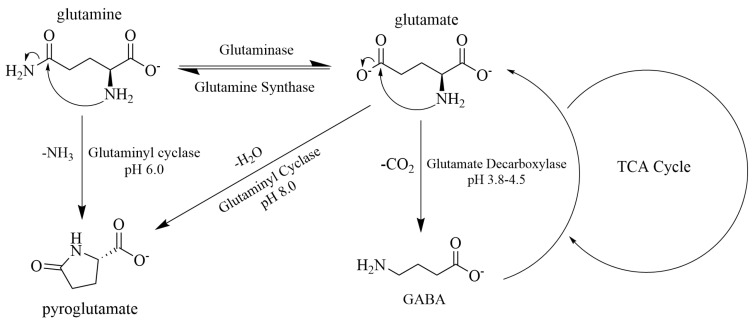
Conversion between glutamatergic metabolites. Abbreviations: TCA, tricarboxylic acid cycle; NH_3_, ammonia; CO_2_, carbon dioxide; GABA, γ-aminobutyric acid; glutamine’s conversion to glutamate is catalyzed via deamidation by glutaminase. The reaction is reversible via amidation of glutamate by glutamine synthase. Glutaminyl cyclase (QC) performs ideally around physiological pH and produces pyroglutamine and pyroglutamate from glutamine and glutamate, respectively, with pyroglutamate being the dominant species. Cyclization and deamination of glutamine forms pyroglutamate. Cyclization and dehydration of glutamate produces pyroglutamate. Decarboxylation of glutamate via glutamate decarboxylase under acidic conditions produces GABA. GABA also produces α-ketoglutarate as a byproduct to feed into the TCA cycle and eventually replenish GABA. This figure was drawn using ChemDraw 23.1.1 64-bit.

Aside from Aβ_42_ plaque and tau tangle aggregation, there is growing interest in whether deficient or excessive neurotransmitter concentrations promote disease progression. A significant neurotransmitter in AD pathology is glutamate (Glu) since excessive extracellular Glu induces excitotoxicity via reactive oxidative species (ROS) release, free radical formation, and mitochondrial dysfunction. At the same time, insufficient concentrations correspond to mental exhaustion [12,13]. Additionally, γ-aminobutyric acid (GABA), glutamine (Gln), and pyroglutamate (PGlu) are also crucial biomolecules derived from glutamate (Figure 1). GABA, a prominent inhibitory neurotransmitter, elevates nitric oxide production in early AD and accompanies tau-induced neurodegeneration [14,15,16]. Gln bolsters the immune system, produces nicotinamide adenine dinucleotide phosphate hydrogen (NADPH), and promotes antioxidant (e.g., glutathione) production [17,18]. Gln and Glu can both be converted into PGlu via Glutaminyl Cyclase (QC) (Figure 1) [17,19,20]. Conversion to PGlu is significant due to the metabolite’s association with deficits in special memory, working memory, and motor function [21]. Thus, the regulation of these biomolecules is needed for optimal cognitive processing. Nevertheless, how can glutamatergic differences relate to the cognitive function of healthy older adults with different amyloid/tau pathology?

One answer could involve frequency band analysis measured using an electroencephalogram (EEG). The electrical brain activity recorded from an EEG is categorized into frequency bands, measured in Hz, as follows: gamma (30–200 Hz), beta (14–30 Hz), alpha (7–13 Hz), theta (4–7 Hz), and delta (0.1–4 Hz), with Hz ranges varying depending on the study [22]. Of these frequency bands, the alpha band, which is the dominant frequency, has been extensively studied for its relevant clinical implications. For example, among all frequency power analyses, EEG alpha band power changes have been related to subtle cognitive dysfunction relative to the risk of cognitive decline [22,23,24,25]. The parietal–occipital distribution of alpha band activity, especially alpha ERD, has been widely studied as correlates of neuronal excitability and attention-orienting behavior during information processing [26]. Alpha ERD is the percent band power change during task presentation relative to the baseline alpha band power measurements [27]. For example, more negative alpha ERD relates to greater brain activation [27]. Prior studies demonstrated positive correlations between Glu levels and resting, low alpha activity in the central, parietal, and frontal brain regions of young (22–30-year-old) CH participants [28,29]. Event-related EEG recordings (i.e., auditory stimulation) in a similar age range of CH individuals produced similar positive correlations between NMDAR (i.e., glutamate receptors) activity and gamma band power [30,31] while other studies produced negative correlations between NMDAR and gamma band power [32,33,34]. These studies place the current understanding of glutamate and its metabolites within a highly varied, electrophysiological context. To our knowledge, the relationships between glutamatergic metabolites and EEG signals have not been studied in cognitively healthy older adults with a higher risk of cognitive decline during cognitive processing [35].

Our study aimed to determine associations between the glutamatergic pathway and changes in cerebral alpha power during WM and Stroop interference tasks as the two core executive functions. We hypothesized that, compared with CH-NATs during Stroop and 0-back WM testing, CH-PATs will have alterations within the glutamatergic pathway corresponding to alpha ERD differences underlying subtle executive dysfunctions. 

## 2. Materials and Methods

### 2.1. Participants

Cognitively healthy participants were recruited from newspaper advertisements, the Pasadena Huntington Hospital Senior Health Network, and the Pasadena Senior Center [25]. The inclusion and exclusion criteria have been described in detail [2]. All participants signed a consent form (HMRI #33797, Quorum IRB, Seattle, Study #27197). An extensive neuropsychological evaluation, including a Mini-Mental State Examination (MMSE) and Montreal Cognitive Assessment (MoCA), was performed to confirm cognitively healthy participants [2,36]. Cerebrospinal fluid (CSF) was collected from each participant via a lumbar puncture [25]. CSF amyloid Aβ_42_, total-tau, and Aβ_42_/total tau ratio were reported [24,25]. Data from 16 CH participants during working memory (WM) testing and 12 CH participants during Stroop interference testing were measured and analyzed.

### 2.2. Working Memory, Stroop Interference Tests, Functional EEG, and Alpha ERD

Cognitive challenges (working memory and Stroop interference tests), EEG collection, and alpha ERD analysis were described [24,25,37]. For N-back, letters were shown on the computer screen one at a time. When the letter was the same as the “N” letter ago, the participant was requested to press “1”, otherwise, press “0”. Each workload condition utilized 3 blocks of 30 trials with ~20 min depending on the participant’s performance. For the Stroop interference test, the words ‘Red’, ‘Blue’, or ‘Green’ were presented on the screen one at a time with two different trial conditions. In congruent trials, the word and ink of the word matched (e.g., ‘Red’ in red ink), while incongruent trials had mismatched words and inks (e.g., ‘Red’ in blue ink). Participants were instructed to conduct practice runs of the Stroop test for 2–3 min. Each test included 3 blocks of 110 trials, resulting in a total test duration of around 20 min and performed in the same data collection room for all participants. 

A 21-head sensor dry electrode system (Quasar Wearable Sensing, DSI-24, San Diego, CA, USA) was placed according to the international 10–20 system for EEG data collection. 

For alpha ERD analysis, all datasets were processed in EEGLAB version eeglab14_l_0b running in MATLAB R2016b (The MathWorks, Natick, MA, USA) and with custom codes developed in-house. We filtered epochs between 2 and 30 Hz, re-referenced EEG data to the mean of 2 mastoid sensors (M1 and M2), and independent component analysis (ICA) was performed to remove artifacts (e.g., eye blinks, cardiac, and muscle activity) [25]. For time-frequency analysis, epoched EEG data were simplified with logarithmic scaling between 2 and 30 Hz by fast Fourier transform and Morlet wavelet convolution in the frequency domain, followed by the inverse fast Fourier transform [25]. Alpha ERD was calculated by normalizing alpha power during task performance to the baseline alpha power before the stimulus onset [dB power = 10*log10(power/baseline)] [25]. Alpha ERD responses were extracted to compare sensors and participant groups [25]. For alpha ERD, we averaged alpha ERD values over 6 brain regions: frontal (Fz, F3, F4), central (Cz, C3, C4), parietal (Pz, P3, P4), left temporal (F7, T3, T5), right temporal (F8, T4, T6), and occipital (O1, O2).

### 2.3. LC Tandem Mass Spectrometry of Plasma Glutamatergic Metabolites

Glutamate (Glu), Glutamine (Gln), Pyroglutamate (PGlu), and γ-aminobutyric acid (GABA) standards were derivatized using an Ez:Faast kit and analyzed in the mass spectrometer, identifying the fragmentations and major peaks of each amino acid (Table 1). The most intense product ions were used for selected reaction monitoring (SRM) in the LC-MS/MS studies. LC-MS/MS quantification of neurotransmitters and amino acids was in accordance with prior studies [32]. Standard solutions of Gln, Glu, PGlu, GABA, and internal standards (homoarginine and homophenylalanine) were made and diluted. After derivatization, the precursor ions and the major product ions of each amino acid were determined by infusion into a triple quadrupole mass spectrometer (TSQ-Quantum, Thermo Fisher, San Jose, CA, USA). Using the LC/MS, a standard curve was obtained for each amino acid and used to determine the concentration of each of the amino acids in plasma samples. The plasma (200 µL) samples were derivatized, and the peak intensity of each amino acid was divided by the intensity of the internal standard to obtain a ratio. The ratio of each amino acid was used to determine their concentration using the linear relationship from the standard curve for each amino acid [32].

### 2.4. Statistical Methods

Group differences from baseline measurements were conducted using two-sided *t*-tests for numerical variables and Fisher exact tests for nominal or categorical variables. *p*-values of demographic data and baseline EEG or metabolite measurements were generated using non-parametric multiple Mann–Whitney U tests within GraphPad Prism 10.1.2. A significance level threshold of 0.05 was used for all tests. Pearson’s correlation coefficients and *p*-values between metabolite concentrations and alpha ERD responses were obtained by normalizing the raw data and performing a correlation matrix of the normalized data in GraphPad Prism 10.1.2.

## 3. Results

### 3.1. Demographic and Neuropsychological Data of WM Participants

Demographic and neuropsychological information were summarized in Table 2. Age, gender ratios, years of education, total tau levels, and cognitive status (i.e., MMSE-7) did not differ (*p* > 0.05) between groups. Greater Aβ_42_/Tau ratios (*p* < 0.002) of CH-NATs indicate sufficient clearance of deleterious protein accumulations, while lower Aβ_42_/tau ratios found in CH-PATs indicate deficient peptide clearance. 

The ratio between each glutamatergic metabolite assessed whether conversion between biomolecules proved useful in differences in clinical classifications. Statistical analysis revealed insignificant differences between individual metabolite concentrations and ratios when comparing CH-NATs with CH-PATs (Supplementary Table S1). No distinctions were made between mean NT concentrations or alpha ERD responses when comparing CH-NATs with CH-PATs (Supplementary Tables S1 and S2). 

### 3.2. Working Memory (WM) Results

**Figure 2:**
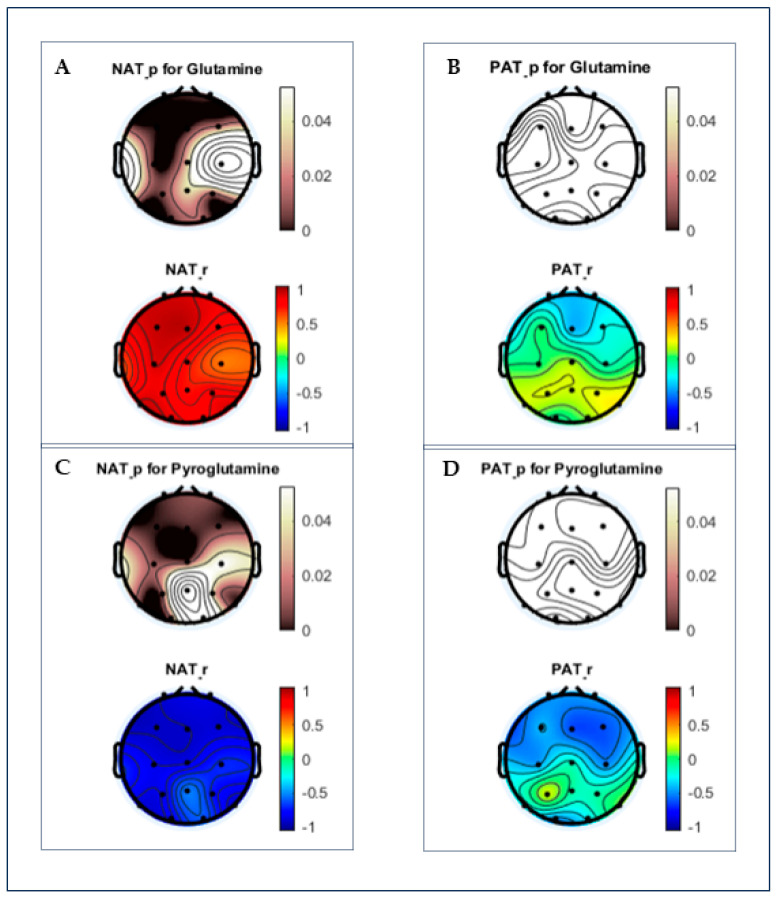
Topoplots of correlations between Glu, Gln, PGlu, and alpha ERD by group. *p*-values (0 to 0.05) are based on a black/white scale, while Pearson correlation coefficients (−1 to 1) are based on the red/green/blue scale. Glutamine concentrations in (**A**) CH-NATs demonstrate prominent central, frontal, and temporal positive correlations (black and red) with alpha ERD, unlike (**B**) insignificant correlations (white) in CH-PATs. Pyroglutamate in (**C**) CH-NATs negatively correlated with frontal, central, and temporal alpha ERD (black and blue), while (**D**) CH-PATs maintained insignificant associations (white).

Under N-back testing, the most prominent outcomes were the correlations (*p* < 0.05) between PGlu, Gln, and alpha ERD responses across all electrode regions. Correlations from CH-NATs suggest greater PGlu concentrations were associated with greater brain activation (i.e., more negative alpha ERD). At the same time, more Gln corresponded to less brain activation (i.e., less negative alpha ERD) (Table 3). These conclusions are supported when the same positive and negative correlations were found in the Gln/PGlu and PGlu/GABA ratios of the F, C, LT, and RT alpha ERD (Table 2). Increasing the Gln/PGlu ratio corresponded to less F, C, LT, and RT activation, and increasing the PGlu/GABA ratio corresponded to greater activation of the same regions. The results were shown in topo-plots by group (Figure 2). 

CH-PATs shared relationships between Glu and GABA levels specific to LT and RT alpha ERD, respectively. In other words, greater Glu and GABA levels corresponded with more pronounced LT and RT activation.

During N-back testing, behavioral responses did not differ between CH-NATs and CH-PATs (Supplementary Table S5), and none of the measured glutamatergic metabolites significantly correlated with behavioral responses (Supplementary Table S6). These findings suggest that the studied metabolites subtly impact EEG alpha band power without grossly influencing participant behavior during a working memory task. 

### 3.3. Stroop Task Results

Stroop participants only differed in Aβ_42/_Tau ratios (*p* < 0.002), which further supports the sufficient waste clearance of harmful Aβ_42_ proteins in CH-NATs (Table 4). 

Although lacking statistical significance, the average alpha ERD responses did differ between CH-NATs and CH-PATs when comparing incongruent to congruent trials (Supplementary Table S4). Greater average global cortical activation was seen in CH-NATs during incongruent testing than in congruent testing (Supplementary Table S4). These findings support the CH-NATs’ resistance to cognitive deterioration as participants allocate optimal cortical activation depending on the task. Unlike CH-NATs, CH-PATs generated closely identical alpha ERD responses during incongruent and congruent testing (Supplementary Table S4). Congruent trials assess participants’ attention, while incongruent trials assess attention and cortical inhibition [38]. Thus, similar alpha responses were expected for CH-PATs in congruent and incongruent task settings to suggest inefficient cortical inhibition. 

During incongruent testing, Gln, Glu, and GABA were notable biomolecules related to cortical activation. Similar to the WM paradigm, Gln shared more prominent relationships when processing stimuli exclusive to CH-NATs. Greater Gln concentrations were associated with greater LT activation and vice versa. Gln/GABA ratios negatively correlated with C (r = −0.97, *p* = 0.027) and LT (r = −0.99, *p* = 0.012) alpha ERD, while PGlu/Glu ratios positively correlated with C (r = 0.98, *p* = 0.021) and LT (r = 0.99, *p* = 0.008) alpha ERD only in CH-NATs (Table 5). A common finding was LT alpha ERD responses associated with Gln, Glu, or GABA. Despite the difference in testing paradigms, WM participants also experienced relationships to LT activation.

During Stroop testing, behavioral responses of CH-PATs differed between their incongruent and congruent trials, while CH-NATs generated insignificant differences between trial responses (Supplementary Table S7). CH-NATs and CH-PATs correlated glutamine concentrations to congruent trial response times and accuracies (Supplementary Table S8). Gln and Gln’s ratio with glutamate positively correlated with CH-PAT response times (r = 0.88, *p* = 0.008 and r = 0.78, *p* = 0.041, respectively). Both CH-NATs and CH-PATs positively correlated between Gln/GABA ratios and accuracy during congruent Stroop trials (r = 0.89, *p* = 0.043 and r = 0.91, *p* = 0.005). 

Many responses to incongruent Stroop testing shared no influence from the measured glutamatergic metabolites. A key finding amongst CH-NATs was the negative correlation between PGlu/Glu and response accuracy during incongruent trials (r = −0.90, *p* = 0.036). The overall findings suggest subtle changes in the EEG and behavioral performance during Stroop task testing can relate to glutamatergic metabolites 

## 4. Discussion

The analysis of our results suggests relationships between glutamine (Gln), pyroglutamate (PGlu), and alpha ERD during cognitive challenges as prominent interests distinguishing cognitively healthy older adults with minimal risk (CH-NAT) from those with elevated risk (CH-PAT) of cognitive decline. The most significant findings from both task-related paradigms were metabolite correlations with ERD in CH-NATs but not CH-PATs. working memory (WM) tests revealed an association of PGlu and Gln to brain activation. Stroop interference testing revealed the significance of Gln to left temporal brain activation. Associations found throughout our study are best explained by looking at glutamatergic metabolism (Figure 1) and considering each metabolite’s contributions to CSF Aβ_42_ or Aβ_42_/tau pathology. 

### 4.1. Working Memory

Under N-back testing, CH-NATs demonstrated a negative correlation between PGlu and alpha ERD across all brain regions (r = ~−0.74–−0.96, *p* = 0.0001–0.041). These correlations suggest that less PGlu corresponds to less brain activation (i.e., less negative alpha ERD) in CH-NATs but not in CH-PATs. Considering the only significant difference between CH-NATs and CH-PATs was the Aβ_42_ ratios and Aβ_42_/tau levels, it is no surprise that PGlu correlates with brain activation in those with adequate CSF clearance. In AD pathogenesis, Aβ peptides undergo N-terminal modification of its Glu residue to form an N-terminal PGlu species of Aβ (Aβ_pE3-42_) peptides catalyzed by glutaminyl cyclase (QC) activity enriched in the temporal (hypothalamus and nucleus basalis) and frontal lobe structures of AD brains [39,40,41]. QC is highly expressed in brain areas affected by AD pathology, including the hippocampus and neocortex [42]. With QC localization in mind, the negative correlations between PGlu concentrations and RT, LT, and F alpha ERD support previous findings. However, the remaining correlations with alpha ERD encourage QC depletion in central, parietal, and occipital brain regions in disease pathogenesis. QC is suggested to facilitate protein stabilization, as seen in its product, Aβ_pE3-42_, having greater resistance to degradation and increased plaque aggregation in AD brains [40,43,44,45]. Less PGlu associated with less brain activation is reasonable considering CH-NATs have more CSF Aβ_42_ (i.e., less brain Aβ) levels and, thus, may effectively eliminate Aβ_pE3-42_ waste.

In contrast, CH-PATs are more vulnerable to degenerative Aβ_pE3-42_ accumulation due to their lower CSF Aβ_42_ (i.e., more brain Aβ) levels. This connection between lower PGlu levels to lower Aβ_42_ and lower brain activation (i.e., less negative alpha ERD) is supported by the higher CSF amyloid/tau ratios seen in CH-NATs. However, under the same correlational relationship, CH-NATs with more brain activation are expected to have higher levels of PGlu despite the limited Aβ_42_ or Aβ_42_/tau burden in CH-NATs’ brains. This finding can be explained by the clearance of Aβ peptides related to the activity of neurotransmitters, such as acetylcholine’s M1 receptor subtype, rather than PGlu presence [46,47,48,49]. Thus, when assessing high-risk older individuals in AD pathogenesis, the transformation of Aβ protein residues into Aβ_pE3-42_ isoforms can be early indicators of cognitive dysfunction detected by the EEG. 

Glutamine positively correlated with alpha ERD in all brain regions (r = ~0.82–0.95, *p* = 0.0003–0.012), meaning greater Gln concentrations corresponded to less brain activation. Unlike PGlu, there is no direct relationship between Gln and Aβ plaque aggregation. However, Gln is the precursor of antioxidants such as glutathione (GSH) [17,18], and American Chemical Society (ACS) reviews detail glutathione’s protection from Aβ-induced oxidative stress in addition to its depletion correlating with MCI and AD classifications [50,51]. Magnetic resonance spectroscopy (MRS) imaging of AD and MCI participants depicted a considerable decrease in GSH in the frontal cortex and hippocampus when compared with age-matched control participants [52,53], with the prominent de-novo GSH synthesis taking place in astrocytes [54]. Thus, Gln’s temporal and frontal distribution can promote greater GSH synthesis to counteract temporal and frontal GSH depletion associated with disease progression. With this perspective, Gln’s positive correlations with alpha ERD in all brain regions may exist to resemble compensatory GSH synthesis to oppose disease onset. 

Gln’s association with decreased brain activation aligns with the previous interpretation of PGlu’s negative correlation with global alpha ERD. Aside from indirect interactions with Aβ_42_ aggregation, the lack of correlations between Gln and brain activation in CH-PATs may raise concerns for an overwhelmed antioxidant system or GSH deficiency [55]. The temporal (hippocampus, amygdala, entorhinal cortex) and frontal lobe structures are vulnerable to oxidative overlap with the brain regions that experience GSH depletion with disease pathogenesis [56,57,58]. These brain regions are especially vulnerable to oxidative damage, aside from GSH insufficiency, due to dependence on oxygen utilization, polyunsaturated fatty acid presence, or metal ion accumulation [59]. The correlations found between Gln and alpha ERD measurements exclusive to CH-NATs support GSH synthesis as fundamental to healthy cognition. Dysregulated oxidation or antioxidant depletion may produce byproducts that inhibit glutamate transporters or activate molecular pathways, releasing nitric oxide in astrocytes and microglia that contribute to Aβ plaque depositions in the hippocampus and cerebral cortex [59]. Thus, glutamine may promote healthy cognition as a GSH precursor. 

Considering the greater CSF Aβ_42_ levels of CH-NATs compared with CH-PATs, it can be speculated that high Gln concentrations also corresponded to lower QC activity and thus reduced conversion of Gln to PGlu (Figure 1). This is supported by postmortem analyses of human temporal cortex samples showing greater QC activity correlated with fewer temporal neurons in AD brains than in age-matched controls [39].

Both interpretations of our correlational data support the contributions of glutamine in CH-NATs to eliminate/resist the Aβ_42_ burden. However, it is unclear how glutamine works to improve healthy cognition: is it via limitation of QC activity or stimulated glutathione production? Evaluating these different mechanisms will make better sense of the correlations gathered under working memory assessments in our study. 

### 4.2. Stroop Task

Stroop task testing shifts the focus from Gln’s antioxidant production to Gln metabolism. Gln’s negative correlation with left temporal alpha ERD (r = −0.96, *p* = 0.037) translates to less Gln associated with less left temporal activation only in CH-NATs. This appears to contradict the findings of the WM task. However, Stroop incongruent trials require the withholding of task responses, so decreased brain activation improves task performance. The existing literature centered around Gln and left temporal brain activity involves reduced Gln as early symptoms for schizophrenia or unrelated to slow wave activity in sleep [60,61]. Thus, the relationship between Gln and LT activation is unclear unless taking into consideration the negative correlation between the Gln/GABA ratio and alpha ERD over the central (r = −0.97, *p* = 0.027) and left temporal (r = −0.97, *p* = 0.027) regions. These correlations mean a decrease in the Gln/GABA ratio corresponding to a decrease in central and left temporal activation (less negative alpha ERD). The localization of glutamate decarboxylase (GAD) isoforms to the neocortex, hippocampus, basal ganglia, and cerebellum of AD brains also makes the corresponding change in LT activity with changes in Gln/GABA ratios reasonable as LT GABA production can be expected from GAD activity [62]. Seeing how both a decrease in the Gln and Gln/GABA ratios is connected to a decrease in LT activity, a shared function between decreased Gln (and increased GABA) could be an explanation. A commonality between Gln and GABA is that the metabolism of both molecules produces α-ketoglutarate, an intermediate in the Krebs’ cycles that produces energy (e.g., ATP) [20,63,64,65,66]. Thus, the correlations between Gln and event-related EEG signaling in CH-NATs suggest that older adults at a reduced risk of cognitive decline can better utilize Gln for energy maintenance during interference processing. Again, validating this hypothesis in a larger sample will be necessary.

Aside from the subtle dysfunction seen by correlations between alpha ERD and metabolite concentrations, incongruent Stroop task accuracy negatively correlated PGlu/Glu ratios. The negative correlation between PGlu/Glu and the accuracy of responses to incongruent Stroop trials implies that greater PGlu concentrations translate to worsened accuracy to incongruent Stroop task demands. This observation aligns with in vivo findings of greater behavioral deficits (i.e., worse performance on balance beams and ataxia) correlating in Aβ_pE3-42_-containing mice [67,68] but without similar findings in human participants. Another interpretation of these behavioral correlations is that higher Glu relative to PGlu translates to more accurate responses to incongruent Stroop trials. The most relevant study connecting this behavioral relationship to Glu was conducted by Biria et al., which positively correlated cortical Glu concentrations in the supplementary motor area with compulsivity metrics [69]. Based on Biria et al.’s study, Glu’s excitatory input may have increased our participants’ responsiveness during interference processing. CH-NATs were more alert when differentiating matched or mismatched color words caused by glutamate’s excitatory function as opposed to CH-PATs experienced reduced arousal most likely because of limited Glu’s excitatory input.

### 4.3. Summarized Findings

Our exploratory study connects brain activation during cognitive challenge tasks (assessed by alpha event-related desynchronization) to glutamine and pyroglutamate due to their relevance in pathogenic Aβ accumulation and energy production. The correlations analyzed under the WM paradigm suggest CH-NATs can appropriately resist/limit the neurodegenerative Aβ_pE3-42_ burden. The analysis during Stroop task testing may suggest CH-NATs demonstrate effective Gln metabolism or GSH synthesis. The biochemical mechanisms responsible for the correlations with the alpha ERD responses remain unclarified. The studied metabolites shared negligible relationships with behavioral outcomes during both EEG recordings. Expanding our findings can further correlate alpha ERD values to structurally similar molecules such as 4-hydroxyproline, asparagine, and aspartate. Analyzing molecules with structural similarities can further isolate whether our electrophysiological findings were specific to glutamatergic metabolites. However, the findings of this study underscore the connection between subtle dysfunction of glutamatergic metabolites and brain activation during cognitive challenge tests (EEG signaling) in cognitively healthy older individuals, which may change with Alzheimer’s pathology. The results need to be validated in a larger population.

### 4.4. Limitations

Our study’s significant limitation was our small sample size. Our pilot study also did not consider known comorbidities. Confounding factors of a bigger population, such as age or sex, can alter the relationships demonstrated by our study. The decrease in alpha power could have also been due to an increase in slow waves or a decrease in faster frequencies. Another limitation is that plasma was not sampled during or after the task performance. This study did not validate the activity of related neurotransmitters or their receptors in the effects of alpha-band responses. Future studies using pharmacologic agents may provide direct knowledge of neurotransmitter–brain activation relationships.

### 4.5. Future Directions

EEG analysis implementation alone improves diagnostic sensitivity and is cost-efficient compared with the current deficiencies of popular imaging techniques. However, the combination of EEG with measurements of neurotransmission and energy metabolites can provide insights into mechanisms and biochemical pathways contributing to EEG differences. Supplementing this study’s findings with measures of Aβ_pE3-42_ can validate or invalidate the proposed involvement of glutamyl cyclase (QC) in the early pathologic conditions of CH-PATs. Calculating the alpha to total EEG power ratio would give more information than relying on alpha ERD measurements, and correlating other frequency band values (delta, beta, gamma, or theta) could provide more electrophysiological insight into the biochemical differences. Moving forward, a longitudinal follow-up supplemented with a greater sample size can elucidate whether the relationships seen in this study persist later in disease pathology. Associational analysis is no definitive marker of causation. Thus, further studies on the crosstalk between neurotransmitter and electrophysiological brain activity can help establish mechanistic underpinnings for disease pathology.

## 5. Conclusions

Our study emphasizes glutamine and pyroglutamate as indications for stable cognitive functionality specific to individuals with normal Aβ_42_/tau pathology (CH-NATs). Working memory tests suggest that less pyroglutamate corresponds to less brain activation, while more glutamine corresponds to less brain activation. This suggests that CH-NATs are more effective in regulating Aβ_42_ from a pathological level than CH-PATs. Correlations found during Stroop task testing suggest less glutamine corresponds to less left temporal and central brain activation, which may be related to performance (by withholding dominant responses) and the potential involvement of energy or antioxidant production during interference task performance. Findings from both WM and Stroop tasks question the relevance of glutaminyl cyclase (QC) activity and Aβ_pE3-42_ accumulation inducing the differences visualized via the EEG signals. A longitudinal follow-up focused on quantifying synaptic transmission and Aβ_pE3-42_ production can elucidate the significance of our findings. In a broader context, EEG and neurotransmitter analysis can offer immense insight into differentiating mechanisms of pathological from normal cognition.

## Figures and Tables

**Table 1:** The molecular weight, derivatized precursor ions.

Amino Acid	MW (g/mol)	Precursor Ion H+ (*m*/*z*)	Products Ions H+ (*m*/*z*)
Gln	146.2	275.3	172, 84.2, 215
Glu	147.1	318	230, 170
PGlu	129.1	172.0	130.0, 84.2
GABA	103.1	232.2	112.2, 200.3
Homoarginine (IS)	188.2	317.0	170.0
Homophenylalanine (IS)	179.2	308.0	117.1

Gln, glutamine; Glu, glutamate; PGlu, pyroglutamate; GABA, γ-aminobutyric acid; IS, internal standards.

**Table 2:** Demographic and Neuropsychological Data of WM Participants.

	CH-NATs	CH-PATs	*p*-Value
Age (yrs.)	75.4 (6.7)	79.1 (7.4)	0.875
Gender (M/F)	2/6	3/5	>0.999 ^#^
Education (yrs.)	16.8 (1.9)	17.6 (2)	0.875
Aβ_42_ Level (pg/mL)	1003.7 (286.0)	421.5 (187.1)	0.004 **
Total Tau Level (pg/mL)	244.1 (78.3)	253.1 (104.3)	0.979
Aβ_42_/Tau	4.6 (1.8)	1.8 (0.6)	0.001 **
MMSE-7	29.1 (1.4)	29.1 (0.8)	0.934

Abbreviations: Aβ_42_, 42-residue beta-amyloid. ^#^ Fisher Exact Test, ** = *p* < 0.005; *p*-values of remaining measurements were gathered from two-tailed *t*-tests. All numbers are recorded as mean (standard deviation).

**Table 3:** Correlations between Alpha ERD and the glutamatergic metabolites in CH-NATs (unshaded) and CH-PATs (shaded) during N-back testing.

Molecule (pg/mL)	F	C	P	LT	RT	O
GABA	r = 0.023 *p* = 0.956	r = 0.18 *p* = 0.672	r = −0.16 *p* = 0.714	r = 0.06 *p* = 0.887	r = 0.14 *p* = 0.746	r = −0.21 *p* = 0.612
	r = −0.76 *p* = 0.028	r = −0.63 *p* = 0.096	r = −0.52 *p* = 0.182	r = −0.44 *p* = 0.277	r = −0.76 *p* = 0.028 *	r = −0.58 *p* = 0.130
PGlu	r = −0.93 *p* = 0.001 **	r = −0.89 *p* = 0.003 **	r = −0.73 *p* = 0.041 *	r = −0.94 *p* = 0.001 **	r = −0.96 *p* = 0.0001 **	r = −0.74 *p* = 0.037 *
	r = −0.61 *p* = 0.109	r = −0.40 *p* = 0.326	r = −0.092 *p* = 0.830	r = −0.42 *p* = 0.295	r = −0.21 *p* = 0.623	r = −0.37 *p* = 0.362
Gln	r = 0.95 *p* = 0.0003 **	r = 0.82 *p* = 0.012 *	r = 0.85 *p* = 0.008 *	r = 0.92 *p* = 0.001 **	r = 0.87 *p* = 0.005 **	r = 0.88 *p* = 0.004 **
	r = −0.43 *p* = 0.297	r = −0.28 *p* = 0.497	r = −0.28 *p* = 0.497	r = −0.28 *p* = 0.504	r = −0.36 *p* = 0.379	r = −0.29 *p* = 0.481
Glu	r = −0.17 *p* = 0.682	r = 0.052 *p* = 0.902	r = −0.21 *p* = 0.614	r = −0.21 *p* = 0.616	r = −0.06 *p* = 0.888	r = −0.43 *p* = 0.291
	r = −0.67 *p* = 0.067	r = −0.58 *p* = 0.129	r = −0.38 *p* = 0.354	r = −0.71 *p* = 0.050 *	r = −0.52 *p* = 0.188	r = −0.50 *p* = 0.206
Gln/Glu	r = 0.28 *p* = 0.502	r = 0.013 *p* = 0.975	r = 0.27 *p* = 0.523	r = 0.26 *p* = 0.542	r = 0.11 *p* = 0.786	r = 0.44 *p* = 0.280
	r = 0.45 *p* = 0.264	r = 0.40 *p* = 0.323	r = 0.12 *p* = 0.775	r = 0.38 *p* = 0.358	r = 0.24 *p* = 0.559	r = 0.34 *p* = 0.414
Gln/PGlu	r = 0.80 *p* = 0.018 *	r = 0.87 *p* = 0.005 **	r = 0.67 *p* = 0.071	r = 0.76 *p* = 0.030 *	r = 0.80 *p* = 0.018 *	r = 0.63 *p* = 0.097
	r = 0.26 *p* = 0.542	r = 0.13 *p* = 0.763	r = −0.27 *p* = 0.521	r = 0.11 *p* = 0.799	r = −0.26 *p* = 0.540	r = 0.074 *p* = 0.861
Gln/GABA	r = 0.73 *p* = 0.038	r = 0.57 *p* = 0.139	r = 0.76 *p* = 0.027	r = 0.70 *p* = 0.052	r = 0.62 *p* = 0.104	r = 0.82 *p* = 0.013 *
	r = −0.13 *p* = 0.760	r = −0.022 *p* = 0.958	r = −0.10 *p* = 0.813	r = −0.091 *p* = 0.831	r = −0.086 *p* = 0.840	r = −0.063 *p* = 0.882
PGlu/Glu	r = −0.49 *p* = 0.219	r = −0.71 *p* = 0.050	r = −0.44 *p* = 0.277	r = −0.53 *p* = 0.179	r = −0.61 *p* = 0.108	r = −0.32 *p* = 0.436
	r = 0.14 *p* = 0.742	r = 0.35 *p* = 0.400	r = 0.52 *p* = 0.184	r = 0.46 *p* = 0.252	r = 0.56 *p* = 0.153	r = 0.27 *p* = 0.518
PGlu/GABA	r = −0.77 *p* = 0.025 *	r = −0.85 *p* = 0.008 *	r = −0.61 *p* = 0.112	r = −0.80 *p* = 0.018 *	r = −0.85 *p* = 0.007 *	r = −0.56 *p* = 0.149
	r = −0.43 *p* = 0.292	r = −0.23 *p* = 0.584	r = 0.053 *p* = 0.902	r = −0.25 *p* = 0.547	r = −0.022 *p* = 0.959	r = −0.23 *p* = 0.585
Glu/GABA	r = −0.080 *p* = 0.851	r = 0.12 *p* = 0.781	r = −0.078 *p* = 0.855	r = −0.12 *p* = 0.774	r = 0.013 *p* = 0.976	r = −0.29 *p* = 0.491
	r = −0.63 *p* = 0.097	r = −0.57 *p* = 0.143	r = −0.36 *p* = 0.384	r = −0.71 *p* = 0.047 *	r = −0.46 *p* = 0.248	r = −0.49 *p* = 0.223

* = *p* < 0.05, ** = *p* < 0.005. Abbreviations: GABA, γ-aminobutyric acid; PGlu, pyroglutamate; Gln, glutamine; Glu, glutamate; F, frontal; C, central; P, parietal; LT, left temporal; RT, right temporal; O, occipital. Pearson’s correlation coefficients (r) and *p*-values are shown for each metabolite and brain region pair.

**Table 4:** Demographic and Neuropsychological Data of Stroop Participants.

	CH-NAT	CH-PAT	*p*-Value
Age (yrs.)	73.2 (2.0)	79.7 (5.8)	0.162
Gender (M/F)	2/3	3/4	>0.999 ^#^
Education (yrs.)	16.8 (0.8)	16.4 (2.1)	0.969
Aβ_42_ Level (pg/mL)	919.6 (249.3)	455.0 (218.7)	0.069
Total Tau Level (pg/mL)	202.2 (62.5)	334.3 (139.9)	0.218
Aβ_42_/Tau	4.7 (1.2)	1.5 (0.6)	0.020 *
MMSE-7	28.8 (1.3)	28.8 (1.0)	>0.999

Abbreviations: Aβ_42_, 42-residue beta-amyloid. ^#^ Fisher Exact Test, * = *p* < 0.05: *p*-values of the remaining measurements were gathered from two-tailed *t*-tests. All numbers were recorded as the mean (standard deviation).

**Table 5:** Correlation Coefficients between Alpha ERD and NTs/AA Concentrations during Incongruent Trials for CH-NATs (unshaded) and CH-PATs (shaded).

Molecule (pg/mL)	F	C	P	LT	RT	O
GABA	r = 0.83 *p* = 0.171	r = 0.61 *p* = 0.386	r = 0.84 *p* = 0.156	r = 0.57 *p* = 0.434	r = 0.86 *p* = 0.144	r = 0.35 *p* = 0.654
	r = −0.08 *p* = 0.871	r = −0.20 *p* = 0.662	r = −0.13 *p* = 0.785	r = −0.35 *p* = 0.443	r = −0.11 *p* = 0.821	r = −0.34 *p* = 0.458
PGlu	r = 0.59 *p* = 0.410	r = 0.81 *p* = 0.195	r = 0.55 *p* = 0.451	r = 0.83 *p* = 0.170	r = −0.48 *p* = 0.517	r = 0.93 *p* = 0.068
	r = −0.54 *p* = 0.215	r = −0.36 *p* = 0.427	r = −0.36 *p* = 0.424	r = −0.34 *p* = 0.457	r = −0.46 *p* = 0.304	r = −0.18 *p* = 0.696
Gln	r = −0.79 *p* = 0.209	r = −0.94 *p* = 0.059	r = −0.71 *p* = 0.289	r = −0.96 *p* = 0.037 *	r = 0.17 *p* = 0.827	r = −0.90 *p* = 0.102
	r = −0.62 *p* = 0.137	r = −0.53 *p* = 0.219	r = −0.62 *p* = 0.135	r = −0.65 *p* = 0.112	r = −0.50 *p* = 0.248	r = −0.52 *p* = 0.231
Glu	r = −0.11 *p* = 0.887	r = 0.14 *p* = 0.863	r = −0.08 *p* = 0.922	r = 0.16 *p* = 0.839	r = −0.87 *p* = 0.128	r = 0.51 *p* = 0.489
	r = 0.15 *p* = 0.744	r = 0.25 *p* = 0.589	r = 0.28 *p* = 0.539	r = 0.17 *p* = 0.717	r = 0.13 *p* = 0.784	r = 0.37 *p* = 0.414
Gln/Glu	r = −0.13 *p* = 0.872	r = −0.42 *p* = 0.581	r = −0.10 *p* = 0.905	r = −0.46 *p* = 0.540	r = 0.84 *p* = 0.156	r = −0.68 *p* = 0.321
	r = −0.25 *p* = 0.593	r = −0.16 *p* = 0.727	r = −0.28 *p* = 0.540	r = −0.24 *p* = 0.602	r = −0.12 *p* = 0.799	r = −0.14 *p* = 0.758
Gln/PGlu	r = −0.80 *p* = 0.198	r = −0.91 *p* = 0.087	r = −0.69 *p* = 0.307	r = −0.94 *p* = 0.061	r = 0.002 *p* = 0.998	r = −0.76 *p* = 0.241
	r = 0.09 *p* = 0.843	r = 0.05 *p* = 0.922	r = −0.02 *p* = 0.973	r = −0.11 *p* = 0.813	r = 0.03 *p* = 0.954	r = −0.12 *p* = 0.801
Gln/GABA	r = −0.87 *p* = 0.134	r = −0.97 *p* = 0.027 *	r = −0.79 *p* = 0.211	r = −0.99 *p* = 0.012 *	r = 0.02 *p* = 0.976	r = −0.88 *p* = 0.118
	r = −0.54 *p* = 0.216	r = −0.41 *p* = 0.366	r = −0.53 *p* = 0.225	r = −0.44 *p* = 0.318	r = −0.39 *p* = 0.382	r = −0.34 *p* = 0.449
PGlu/Glu	r = 0.87 *p* = 0.128	r = 0.98 *p* = 0.021 *	r = 0.80 *p* = 0.197	r = 0.99 *p* = 0.008 *	r = −0.03 *p* = 0.970	r = 0.90 *p* = 0.098
	r = −0.60 *p* = 0.153	r = −0.42 *p* = 0.344	r = −0.48 *p* = 0.275	r = −0.32 *p* = 0.491	r = −0.41 *p* = 0.365	r = −0.25 *p* = 0.593
PGlu/GABA	r = 0.56 *p* = 0.439	r = 0.78 *p* = 0.216	r = 0.52 *p* = 0.481	r = 0.81 *p* = 0.189	r = −0.51 *p* = 0.487	r = 0.92 *p* = 0.080
	r = −0.39 *p* = 0.384	r = −0.18 *p* = 0.698	r = −0.20 *p* = 0.660	r = −0.13 *p* = 0.785	r = −0.30 *p* = 0.510	r = −0.02 *p* = 0.962
Glu/GABA	r = −0.24 *p* = 0.765	r = 0.01 *p* = 0.988	r = −0.19 *p* = 0.805	r = 0.04 *p* = 0.964	r = −0.90 *p* = 0.103	r = 0.40 *p* = 0.600
	r = 0.20 *p* = 0.668	r = 0.33 *p* = 0.469	r = 0.34 *p* = 0.453	r = 0.29 *p* = 0.533	r = 0.18 *p* = 0.698	r = 0.46 *p* = 0.298

* = *p* < 0.05. Abbreviations: GABA, γ-aminobutyric acid; PGlu, pyroglutamate; Gln, glutamine; Glu, glutamate; F, frontal; C, central; P, parietal; LT, left temporal; RT, right temporal; O, occipital. Pearson’s correlation coefficients (r) and *p*-values are shown for each metabolite and brain region pair.

## Data Availability

Data can be provided upon reasonable request.

## Supplementary Materials

The following supporting information can be downloaded at: https://www.mdpi.com/article/10.3390/cells13110970/s1, Table S1: Mean (SD) Glut, Gln, PGlu, GABA Concentrations (pg/mL) of CH-NATs and CH-PATs participating in working memory tests, Table S2: Mean (SD) Alpha ERD responses (dB) during N-back testing, Table S3: Mean (SD) Glut, Gln, PGlu, and GABA concentrations (pg/mL) of CH-NATs and CH-PATs participating in Stroop testing, Table S4: Comparison of Mean (SD) Alpha ERD (dB) between CH-NATs and CH-PATs during congruent and incongruent Stroop task trials, Table S5: Mean (SD) response times and accuracies during N-back testing; Table S6: Correlations between glutamatergic metabolites and behavioral responses in CH-NATs (unshaded) and CH-PATs (shaded) during N-back testing, Table S7: Mean (SD) response times and accuracies during Stroop task testing, Table S8: Correlation coefficients between glutamatergic metabolites and behavioral responses during Stroop task testing for CH-NATs (unshaded) and CH-PATs (shaded) during Stroop task testing.
