# Supplementary material for: Associations of Plasma Glutamatergic Metabolites with Alpha Desynchronization during Cognitive Interference and Working Memory Tasks in Asymptomatic Alzheimer’s Disease
Source: Cells. 2024 Jun 4;13(11):970. doi: 10.3390/cells13110970 (PMC11171970; doi:10.3390/cells13110970)
Supplement: Supplementary file 1 [file cells-13-00970-s001.zip › cells-2986745-supplementary.pdf]

5. Supplementary Materials

Table S1: Mean (SD) Glut, Gln, PGlu, GABA Concentrations (pg/mL) of CH-NATs and CH-PATs participating in working memory tests.

Molecule (pg/mL)	CH-NATs	CH-PATs	p-value
GABA	0.083 (0.015)	0.082 (0.016)	0.959
PGlu	0.70 (0.62)	1.20 (0.88)	0.898
Gln	71.36 (27.41)	85.84 (51.52)	0.910
Glu	42.49 (24.39)	34.16 (33.55)	0.793
Gln/Glu	1.68 (1.29)	2.51 (3.03)	0.908
Gln/PGlu	101.82 (128.85)	71.63 (155.37)	0.910
Gln/GABA	863.33 (365.06)	1042.99 (547.87)	0.910
PGlu/Glu	0.02 (0.04)	0.04 (0.02)	0.188
PGlu/GABA	8.48 (9.49)	14.56 (9.99)	0.910
Glu/GABA	514.04 (270.35)	415.03 (322.39)	0.882

Abbreviations: GABA, γ-aminobutyric acid; PGlu, pyroglutamate; Gln, glutamine; Glu, glutamate; P-values obtained using multiple Mann-Whitney U tests compared metabolite concentrations between CH-NATs and CH-PATs.

Table S2: Mean (SD) Alpha ERD Responses (dB) during N-back testing

Electrode Region	CH-NATs	CH-PATs	p-values
F	-0.63 (1.27)	-1.50 (1.41)	0.580
C	-0.55 (1.04)	-1.57 (1.03)	0.332
P	-1.26 (1.20)	-1.85 (1.08)	0.580
LT	-0.73 (1.21)	-1.65 (0.93)	0.352
RT	-0.64 (1.36)	-1.72 (1.22)	0.580
O	-0.89 (1.01)	-1.15 (0.91)	0.580

Abbreviations: F, frontal region; C, central region; P, parietal region; LT, left temporal region; RT, right temporal region; O, occipital region. Alpha ERD responses were measured in decibels (dB). P-values obtained using multiple Mann-Whitney U tests compared alpha ERD responses between CH-NATs and CH-PATs.

Table S3: Mean (SD) Glut, Gln, PGlu, and GABA Concentrations (pg/mL) of CH-NATs and CH-PATs participating in Stroop testing.

Molecule (pg/mL)	CH-NATs	CH-PATs	p-value
GABA	0.095 (0.015)	0.081 (0.016)	0.887
PGlu	0.74 (0.66)	0.85 (0.50)	0.985
Gln	78.42 (39.03)	75.20 (44.77)	>0.999
Glu	44.43 (17.51)	23.69 (12.32)	0.388
Gln/Glu	2.20 (1.22)	4.27 (2.97)	0.836
Gln/PGlu	211.28 (158.43)	155.67 (158.14)	0.977
Gln/GABA	883.27 (500.99)	950.58 (573.17)	0.985
PGlu/Glu	0.016 (0.0084)	0.037 (0.020)	0.496
PGlu/GABA	7.86 (6.45)	11.14 (6.90)	0.983
Glu/GABA	494.63 (216.60)	300.53 (170.95)	0.887

Abbreviations: GABA, γ-aminobutyric acid; PGlu, pyroglutamic acid; Gln, glutamine; Glu, glutamate. P-values obtained using multiple Mann-Whitney U tests compared metabolite concentrations between CH-NATs and CH-PATs.

Table S4: Comparison of Mean (SD) Alpha ERD (dB) Between CH-NATs and CH-PATs During Congruent and Incongruent Stroop Task Trials

Electrode Position	CH-NATs		p-value	CH-PATs		p-value
	Congruent	Incongruent		Congruent	Incongruent	
F	-0.94 (0.24)	-1.83 (1.01)	0.571	-1.41 (1.09)	-1.39 (1.19)	>0.999
C	-0.94 (0.30)	-1.98 (0.89)	0.571	-1.58 (1.37)	-1.47 (1.25)	>0.999
P	-0.98 (0.35)	-2.08 (0.71)	0.0915	-1.63 (0.99)	-1.61 (1.29)	>0.999
LT	-0.81 (0.20)	-1.79 (1.02)	0.571	-1.44 (0.99)	-1.47 (1.04)	>0.999
RT	-0.73 (0.55)	-2.01 (0.31)	0.0915	-1.44 (0.87)	-1.48 (1.11)	>0.999
O	-1.30 (0.46)	-1.89 (0.42)	0.571	-1.74 (0.87)	-1.74 (1.18)	>0.999

Abbreviations: F, frontal region; C, central region; P, parietal region; LT, left temporal region; RT, right temporal region; O, occipital region. Alpha ERD responses were measured in decibels (dB). P-values generated from non-parametric, Multiple Mann-Whitney U tests were used to compare alpha ERD responses between incongruent and congruent trials in CH-NATs and CH-PATs.

Data from 4 participants during incongruent testing of the CH-NAT group was measured and analyzed, as opposed to the expected 5 participants, due to missing EEG data from incongruent trial testing for one CH-NAT.

Table S5: Mean (SD) Response Times and Accuracies during N-back Testing

Response Category	CH-NATs	CH-PATs	p-value
Time	579.48 (108.39)	540.22 (61.46)	0.574
Accuracy	0.90 (0.06)	0.90 (0.08)	0.857

P-values using Mann-Whitney U tests compared response times and accuracies between CH-NATs and CH-PATs.

Table S6: Correlations between glutamatergic metabolites and behavioral responses in CH-NATs (unshaded) and CH-PATs (shaded) during N-back Testing

Molecule (pg/mL)	Accuracy	Response Time
GABA	r= -0.66 p=0.074	r=0.14 p=0.739
	r= -0.53 p=0.181	r=0.13 p=0.768
PGlu	r=0.11 p=0.787	r=0.42 p= 0.303
	r=0.20 p=0.641	r=0.48 p=0.231
Gln	r=0.29 p=0.485	r= -0.35 p= 0.398
	r= -0.11 p=0.802	r=0.52 p=0.185
Glu	r=0.17 p=0.686	r= 0.24 p= 0.574
	r=0.15 p=0.716	r=0.67 p=0.068
Gln/Glu	r=0.21 p=0.622	r= -0.29 p=0.484

	r=0.02 p=0.961	r=0.03 p=0.946
Gln/PGlu	r= -0.16 p=0.706	r= -0.29 p=0.488
	r= -0.12 p=0.776	r=0.08 p=0.854
Gln/GABA	r=0.55 p=0.162	r= -0.30 p=0.466
	r=0.10 p=0.822	r=0.55 p=0.155
PGlu/Glu	r=0.46 p=0.250	r= -0.10 p=0.811
	r=0.22 p=0.608	r= -0.18 p=0.666
PGlu/GABA	r=0.36 p=0.381	r=0.19 p=0.647
	r=0.32 p=0.443	r=0.35 p=0.394
Glu/GABA	r=0.34 p=0.404	r=0.19 p=0.654
	r=0.21 p=0.613	r=0.63 p=0.095

* = p<0.05

Abbreviations: GABA, γ-aminobutyric acid; PGlu, pyroglutamate; Gln, glutamine; Glu, glutamate. Pearson's correlation coefficients (r) and p-values are shown for each metabolite and behavioral response.

Table S7: Mean (SD) Response Times and Accuracies during Stroop Task Testing

	CH-NATs		p-value	CH-PATs		p-value
Response Category	Congruent	Incongruent		Congruent	Incongruent	
Time	724.40 (168.68)	870.33 (207.79)	0.0625	779.49 (109.65)	1030.68 (128.30)	0.0156
Accuracy	0.92 (0.04)	0.90 (0.04)	0.3750	0.93 (0.03)	0.85 (0.09)	0.0781

P-values generated from non-parametric, unpaired non-parametric, Mann-Whitney U tests were used to compare response times and accuracies between congruent and incongruent Stroop tasks in CH-NATs and CH-PATs.

Table S8. Correlation Coefficients Between Glutamatergic Metabolites and Behavioral Responses During Stroop Task Testing for CH-NATs (unshaded) and CH-PATs (shaded) during Stroop Task Testing

	Congruent		Incongruent	
Molecule (pg/mL)	Accuracy	Response Time	Accuracy	Response Time
GABA	r= -0.76 p=0.139	r=0.43 p=0.473	r= -0.46 p=0.440	r=0.20 p=0.747
	r=0.18 p=0.700	r=0.71 p=0.075	r= -0.43 p=0.342	r= -0.19 p=0.679
PGlu	r= -0.70 p=0.187	r=0.25 p=0.683	r= -0.79 p=0.115	r=0.23 p=0.704
	r= -0.12	r= -0.67	r= -0.26	r=0.44

	p=0.804	p=0.097	p=0.571	p=0.329
Gln	r=0.76<br>p=0.135	r= -0.49<br>p=0.401	r=0.85<br>p=0.066	r= -0.45<br>p=0.443
	r=0.07<br>p=0.890	r=0.88*<br>p=0.008*	r= -0.06<br>p=0.906	r=0.59<br>p=0.163
Glu	r= -0.25<br>p=0.682	r= -0.40<br>p=0.501	r= -0.20<br>p=0.743	r=-0.39<br>p=0.522
	r= -0.54<br>p= 0.216	r= 0.50<br>p= 0.250	r= -0.65<br>p= 0.114	r=0.02<br>p=0.963
Gln/Glu	r=0.38<br>p=0.524	r=0.23<br>p=0.712	r=0.39<br>p=0.516	r=0.19<br>p=0.763
	r=0.41<br>p=0.365	r=0.78<br>p=0.041*	r=0.12<br>p=0.793	r=0.53<br>p=0.223
Gln/PGlu	r=0.39<br>p=0.515	r= -0.59<br>p=0.291	r=0.72<br>p=0.167	r= -0.67<br>p=0.218
	r=0.66<br>p=0.109	r= -0.37<br>p=0.415	r= -0.03<br>p=0.953	r=0.01<br>p=0.991
Gln/GABA	r=0.89<br>p=0.043*	r= -0.49<br>p=0.397	r=0.82<br>p=0.093	r= -0.38<br>p=0.531
	r=0.91<br>p=0.005*	r=0.31<br>p=0.502	r=0.19<br>p=0.679	r=0.65<br>p=0.114
PGlu/Glu	r= -0.76<br>p=0.137	r=0.61<br>p=0.271	r= -0.90<br>p=0.036*	r=0.57<br>p=0.315
	r=0.72<br>p=0.071	r= -0.27<br>p=0.560	r=0.21<br>p=0.653	r=0.66<br>p=0.105
PGlu/GABA	r= -0.57<br>p=0.316	r=0.23<br>p=0.711	r= -0.74<br>p=0.155	r=0.25<br>p=0.680
	r= -0.54<br>p=0.210	r= -0.01<br>p=0.986	r= -0.16<br>p=0.738	r=0.51<br>p=0.245
Glu/GABA	r=0.13<br>p=0.832	r= -0.41<br>p=0.488	r=0.00<br>p=0.995	r= -0.30<br>p=0.618
	r=0.01<br>p=0.989	r= -0.63<br>p=0.127	r= -0.52<br>p=0.227	r=0.08<br>p=0.867

* = p<0.05

Abbreviations: GABA, γ-aminobutyric acid; PGlu, pyroglutamate; Gln, glutamine; Glu, glutamate. Pearson's correlation coefficients (r) and p-values are shown for each metabolite and behavioral response.
